# Intermittent hypoxia‐induced epiregulin expression by IL‐6 production in human coronary artery smooth muscle cells

**DOI:** 10.1002/2211-5463.12430

**Published:** 2018-04-19

**Authors:** Yoji Kyotani, Asako Itaya‐Hironaka, Akiyo Yamauchi, Sumiyo Sakuramoto‐Tsuchida, Mai Makino, Shin Takasawa, Masanori Yoshizumi

**Affiliations:** ^1^ Department of Pharmacology Nara Medical University Kashihara Japan; ^2^ Department of Biochemistry Nara Medical University Kashihara Japan

**Keywords:** epiregulin, interleukin‐6, intermittent hypoxia

## Abstract

Patients with obstructive sleep apnea (OSA) experience repetitive episodes of desaturation and resaturation of blood oxygen (known as intermittent hypoxia or IH), during sleep. We showed previously that IH induced excessive proliferation of rat vascular smooth muscle cells through upregulation of members of the epidermal growth factor family, especially epiregulin (EREG), and the erbB2 receptor. In this study, we exposed human coronary artery smooth muscle cells to IH and found that IH significantly increased the expression of EREG. IH increased the production of interleukin‐6 (IL‐6) in smooth muscle cells, and the addition of IL‐6 induced EREG expression. Small interfering RNA for IL‐6 or IL‐6 receptor attenuated the IH‐induced increase in EREG. IL‐6 may play a pivotal role in EREG upregulation by IH and consequently OSA‐related atherosclerosis.

**Abbreviations**
AREG: amphiregulin
CPAP: continuous positive airway pressure
EGF: epidermal growth factor
EREG: epiregulin
hCASMC: human coronary artery smooth muscle cell
IH: intermittent hypoxia
IL‐6: interleukin‐6
NFκB: nuclear factor‐kappa B
OSA: obstructive sleep apnea
RASMC: rat aorta smooth muscle cell
SH: sustained hypoxia
VSMC: vascular smooth muscle cell

We previously reported that intermittent hypoxia (IH) induced the proliferation of cultured rat aorta smooth muscle cells (RASMCs) through upregulation of members of the epidermal growth factor (EGF) family, including epiregulin (EREG), amphiregulin (AREG), and neuregulin‐1, and the erbB2 receptor 1. The role of IH in obstructive sleep apnea (OSA), which is an independent risk factor for cardiovascular and other diseases, is well known 2, 3, 4, 5, 6. In the progression of atherosclerosis, which is a major cause of cardiovascular events, inflammation, foam‐cell formation, and excessive proliferation of vascular smooth muscle cell (VSMC) are most important properties. Thus, macrophages recruited to vessel wall by inflammation take in the oxidized aggregated low‐density lipoprotein via scavenger receptors resulting in foam‐cell formation and express cytokines to influence VSMC growth and matrix accumulation, which give rise to a fibrous cap 7. For prophylaxis against OSA‐related diseases, continuous positive airway pressure (CPAP) is applied in a clinical setting. Various studies have demonstrated dramatic effects of prophylactic CPAP on atherosclerosis and cardiovascular mortality 8, 9, 10, 11. However, its compliance with treatment for OSA such as CPAP is often unsatisfactory 12, 13, 14. Studies of cellular responses to IH could establish another prophylactic approach to combat OSA‐related diseases.

Interleukin‐6 (IL‐6) is a proinflammatory cytokine, which plays an important role in acute and chronic inflammation. We previously reported that IH stimulated pancreatic β cells to induce IL‐6 gene expression 15. Several studies reported that serum inflammatory markers, including IL‐6, were increased in OSA patients 16, 17, 18. In addition, IL‐6 induced an increase in EREG mRNA expression in mouse endothelial cells 19. On the basis of the aforementioned findings, we speculated that IL‐6 may be a key molecule in OSA‐related diseases such as atherosclerosis. In this study, using human coronary artery smooth muscle cells (hCASMCs), we investigated the influence of IH on IL‐6 production and the association of IL‐6 with IH‐induced increases in EREG mRNA expression.

## Materials and methods

### Cell culture

SV40‐immortalized hCASMCs were purchased from Applied Biological Materials Inc. (Richmond, BC, Canada) and grown in medium (Prigrow II/III) containing 5% (v/v) fetal bovine serum, as specified by the supplier. The cells were maintained at atmospheric oxygen concentrations (21% O_2_, 5% CO_2_; 37 °C; normoxia). To produce sustained hypoxia (SH), the cells were maintained in a hypoxia chamber (1% O_2_, 5% CO_2_; balance N_2_ and water vapor). To induce IH, the cells were exposed to cycles of hypoxic (5 min) and normoxic (10 min) conditions as described previously 1, 15, 20, 21, 22. The cells exposed to normoxia, SH, or IH were cultured under the conditions described above.

### Real‐time RT‐PCR assay

Total RNA was extracted from hCASMCs using an RNeasy Protect Cell Mini Kit (Qiagen, Hilden, Germany), according to the manufacturer's protocol, as described previously 1, 20, 21, 22, 23, 24. After quantifying the isolated RNA using a spectrophotometer, 0.5‐μg aliquots were reverse‐transcribed using a High‐Capacity cDNA Reverse Transcription Kit (Applied Biosystems, Foster City, CA, USA). PCR primers were obtained from Nihon Gene Research Laboratories (Sendai, Japan). The primer sequences used to amplify IL‐6, EREG, and AREG mRNA are shown in Table 1. The real‐time PCR assay was carried out using a SYBR® qPCR kit (KAPA Biosystems, Woburn, MA, USA) in a Thermal Cycler Dice (TaKaRa, Kusatsu, Japan). The mRNA expression level was normalized to that of β‐actin.

**Table 1:** Primers used for RT‐PCR

Target gene	Primer sequence (position)
IL‐6	5′‐ GGTACATCCTCGACGGCATC‐3′ (NM_000600: 289–308)
	5′‐ GCCTCTTTGCTGCTTTCACAC‐3′ (NM_000600: 347–367)
EREG	5′‐ CAAAGTGTAGCTCTGACATG‐3′ (NM_001432: 363–382)
	5′‐ CTGTACCATCTGCAGAAATA‐3′ (NM_001432: 581–600)
AREG	5′‐ TGCTGGATTGGACCTCAATG‐3′ (NM_001657: 320–339)
	5′‐ TCCCGAGGACGGTTCACTAC‐3′ (NM_001657: 463–482)
β‐Actin	5′‐GCGAGAAGATGACCCAGA‐3′ (NM_001101: 420–437)
	5′‐CAGAGGCGTACAGGGATA‐3′ (NM_001101: 492–509)

### Promoter assay

A promoter construct was prepared by inserting a fragment of human EREG gene (−1345 to +118: NC_018915) upstream of a firefly luciferase reporter gene pGL4.17[*luc*/Neo] vector (Promega, Madison, WI, USA). The cells were grown in 24‐well plates to 70–80% confluency and transfected with a reporter plasmid by lipofection using Lipofectamine® 3000 (Life Technologies, Carlsbad, CA, USA), as described previously 22, 23, 24. After IH, SH, or normoxia treatment for 24 h, the cells were harvested, and cell extracts were prepared in extraction buffer [0.1 m potassium phosphate (pH 7.8)/0.2% Triton X‐100; Life Technologies]. To monitor the transfection efficiency, a pCMV‐SPORT‐βgal plasmid (Life Technologies) was cotransfected in all experiments at a 1 : 10 dilution. Luciferase activity was measured using the PicaGene luciferase assay system (Toyo Ink, Tokyo, Japan) and normalized by β‐galactosidase activity, as previously described 20, 21, 22, 23, 24, 25.

### Measurement of IL‐6 and EREG expressions in IH‐exposed hCASMC cultured medium

**Figure 1:**
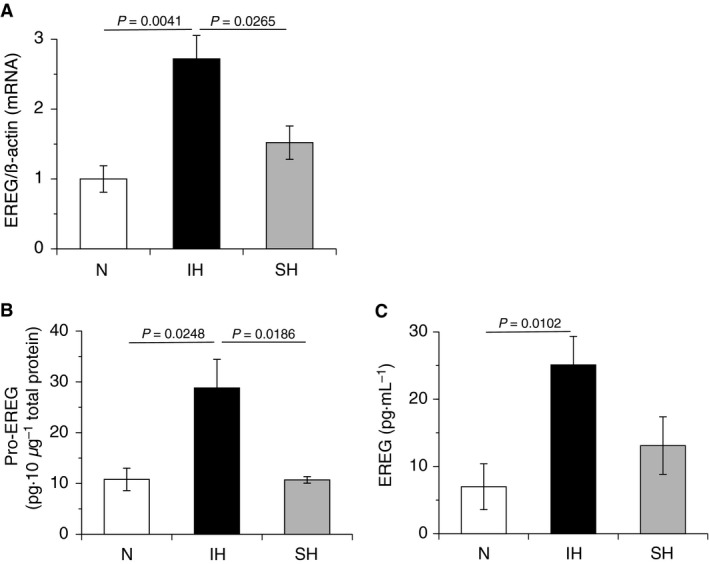
IH increased the expression of EREG in hCASMCs. hCASMCs were cultured for 24 h with serum‐free medium and exposed to normoxia, IH, or SH for 24 h. (A) Total RNA were extracted, and real‐time RT‐PCR was performed using specific primers for human EREG mRNA, as described in the Materials and methods section. Each value was normalized by arbitrarily setting the value of β‐actin of the cells exposed to normoxia to 1.0. The results are representative of four independent experiments. (B) Normoxic‐, IH‐, and SH‐treated hCASMCs were denatured by repeats of freeze and thaw. Ten micrograms of total protein in each cell lysate was used in an EREG immunoassay, as described in the Materials and methods section. The results are representative of five independent experiments. (C) Conditioned media of normoxic‐, IH‐, and SH‐treated hCASMCs were collected and used in an EREG immunoassay, as described in the Materials and methods section. The results are representative of four to five independent experiments. Each point represents the mean ± SEM.

**Figure 2:**
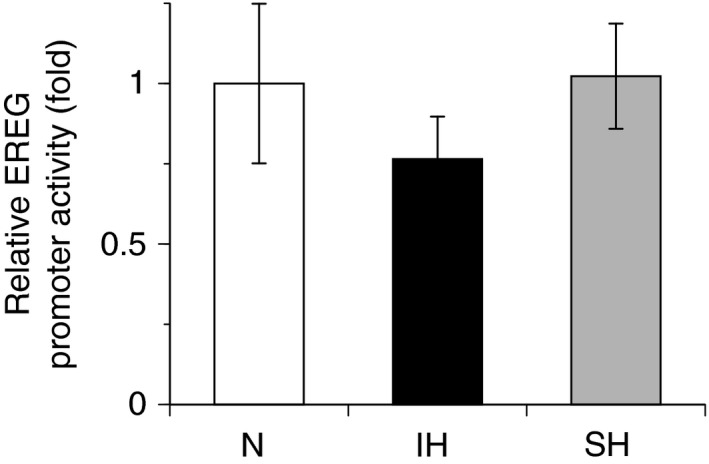
IH has no effect on promoter activity of EREG in hCASMCs. Reporter plasmids prepared by inserting the promoter fragments of EREG (−1345 to +118) upstream of a firefly luciferase reporter gene in the pGL4.17 vector were transfected into hCASMCs. After cells were exposed to 24 h of either IH, SH, or normoxia, the cells were lysed, and promoter activities of EREG were measured, as described in the Materials and methods section. Each value was normalized by arbitrarily setting the value of luciferase/β‐galactosidase activity of the cells exposed to normoxia to 1.0. The results are representative of four independent experiments. Each point represents the mean ± SEM.

Conditioned media and cells were collected to examine the expression of human IL‐6 and EREG after exposure to IH for the indicated times (Fig. 1B,C, 2C, and 4B). IL‐6 and EREG were detected by a human IL‐6 immunoassay (R&D Systems, Minneapolis, MN, USA) and human EREG immunoassay (LifeSpan BioSciences, Inc., Seattle, WA, USA), respectively, according to the manufacturer's instructions. Briefly, cell lysates were prepared by repeats of freeze and thaw, and the protein assay was performed using coomassie brilliant blue solution. Conditioned media or cell lysates (10 μg of total protein) were added into a 96‐well plate, which was precoated with a monoclonal antibody specific for human IL‐6 or EREG. After washing away any unbound materials, an enzyme‐linked antibody specific for human IL‐6 or EREG was added to the wells. Following washing to remove any unbound antibody/enzyme reagent, a substrate solution was added to the wells. The intensity of the light emitted was measured by a microplate luminometer (POWERSCAN® HT; BioTek Instruments, Inc., Winooski, VT, USA).

### RNA interference (RNAi)

RNA interference (RNAi) was performed using small interfering RNA (siRNA) directed against human IL‐6 and IL‐6 receptor genes. The sequences of siRNA for IL‐6 and the IL‐6 receptor were 5′‐GGACAUGACAACUCAUCUCtt‐3′ and 5′‐CGACUCUGGAAACUAUUCAtt‐3′, respectively 26. Silencer® Select Negative Control No. 1 siRNA (Thermo Fisher Scientific, Waltham, MA, USA) was used as a control. Transfection of siRNA to hCASMCs was carried out using Lipofectamine™ RNAiMAX transfection reagent (Thermo Fisher Scientific). The cells were transfected with 10 pmol each of siRNA in a 12‐well culture dish as described previously 15, 22, 23, 24.

### Statistical analysis

All the experiments were performed in triplicate or more. The values obtained are described as means ± SEM. After performing a two‐way ANOVA to determine the significance among groups, a modified *t*‐test was conducted, with Fisher's post hoc test performed for intergroup comparisons. A *P* value of < 0.05 was considered statistically significant.

## Results

### IH increased the expression of EREG in hCASMCs

We first investigated whether EREG expression increased in response to IH stimulation in hCASMCs, as previously observed in RASMCS 1. As shown in Fig. 1A, EREG mRNA expression increased in response to IH but not SH in hCASMCs, as previously reported in RASMCs 1. The EREG functions in an autocrine fashion. Thus, transmembrane EREG proform (pro‐EREG) is cleaved and released into extracellular space as mature form of EREG. In additional experiments, pro‐EREG in cell lysate and EREG in cell conditioned medium also increased in response to IH but not SH (Fig. 1B,C). These results indicate that the IH‐induced increase in EREG mRNA correlates with increases in pro‐EREG and EREG and that upregulation of EREG in response to IH is a common feature of VSMCs.

### IH‐induced gene expression of EREG was not directly regulated by transcription

To determine whether the IH‐induced increases in EREG mRNA were caused by activation of transcription, human EREG promoter was fused to the luciferase gene of pGL4.17 and transfected into hCASMCs. IH stimulation did not markedly increase the activity of the EREG promoter (Fig. 2), suggesting that the gene expression of EREG in response to IH was not directly regulated by transcription.

### IH induced IL‐6 expression in a time‐dependent manner

**Figure 3:**
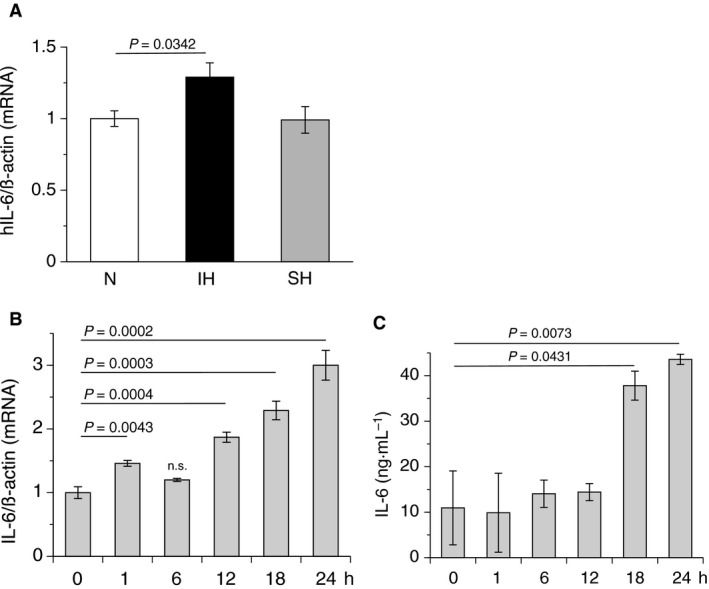
IH induced IL‐6 production in a time‐dependent manner. (A) After exposure of hCASMCs to normoxia, IH, or SH for 24 h, total RNA were extracted, and real‐time RT‐PCR was performed using specific primers for human IL‐6 mRNA, as described in the Materials and methods section. Each value was normalized by arbitrarily setting the value of β‐actin of the cells exposed to normoxia to 1.0. The results are representative of five independent experiments. (B) After exposure of hCASMCs to normoxia, IH, or SH for the indicated times (h) in the body of the figure, total RNA were extracted, and real‐time RT‐PCR was performed using specific primers for human IL‐6 mRNA, as described in the Materials and methods section. Each value was normalized by arbitrarily setting the value of β‐actin of the cells exposed to normoxia (0 h) to 1.0. The results are representative of four independent experiments. IL‐6 mRNA after IH stimulation (1, 12, 18, and 24 h, except 6 h) was significantly increased. ‘n.s.’, not significantly different from 0 h. (C) After exposure of hCASMCs to normoxia, IH, or SH for the indicated times (h) in the body of the figure, conditioned media of normoxic‐, IH‐, and SH‐treated hCASMCs were collected and used in a human IL‐6 immunoassay, as described in the Materials and methods section. The results are representative of four independent experiments. Each point represents the mean ± SEM.

As the involvements of IL‐6 in IH‐induced cellular responses 14, 15, 16, 17, we investigated IL‐6 mRNA expression using real‐time RT‐PCR. As shown in Fig. 3A, the expression of IL‐6 mRNA was increased by IH, but not by SH. In addition, IH significantly increased IL‐6 mRNA expression in a time‐dependent manner, with upregulation observed from 1 h to 24 h (Fig. 3B). Similar increases in IL‐6 were observed in IH‐stimulated RASMCs (data not shown). Additional analyses revealed that mature IL‐6 increased in IH‐exposed cell conditioned medium in a similar time‐dependent manner as seen in mRNA (Fig. 3C). These results are consistent with the rise in blood IL‐6 levels observed in patients with moderate/severe OSA 16, 17, 18.

### IL‐6 stimulus increased EREG mRNA expression

**Figure 4:**
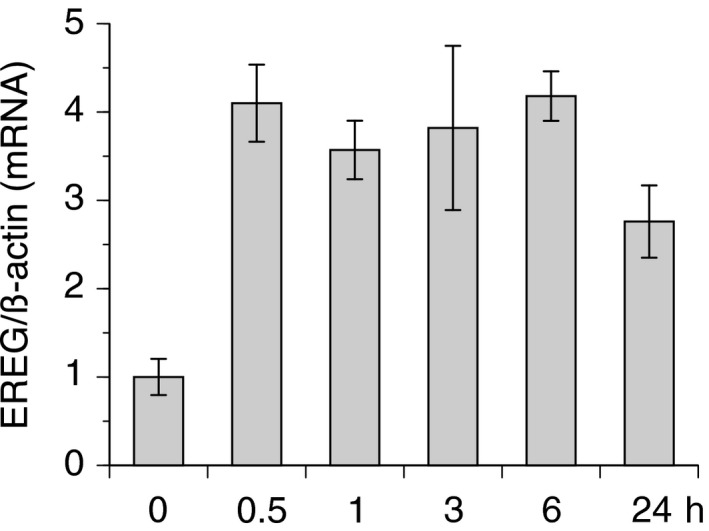
IL‐6 stimulus increased EREG mRNA expression. hCASMCs were cultured for 24 h with serum‐free medium and stimulated with 100 ng·mL^−1^ of IL‐6 for the indicated hours. Then, total RNA were extracted, and real‐time RT‐PCR was performed using specific primers for human EREG mRNA, as described in the Materials and methods section. Each value was normalized by arbitrarily setting the value of β‐actin of the cells exposed to normoxia to 1.0. The results are representative of four independent experiments. Each point represents the mean ± SEM. IL‐6‐induced increase in EREG mRNA in each time point was statistically significant compared with that in 0 h (*P* < 0.05).

Previous studies reported that IL‐6 increased EREG mRNA expression in mouse endothelial cells 19. Thus, we tested whether IL‐6 increased the expression of EREG mRNA in hCASMCs. As shown in Fig. 4, following the addition of 100 ng·mL^−1^ of IL‐6 to the hCASMC cultured medium, EREG mRNA was upregulated from 0.5 to 24 h after stimulation with IL‐6, suggesting that IH‐induced EREG expression was induced by IL‐6 increases in hCASMCs.

### siRNA directed against IL‐6 and the IL‐6 receptor suppressed the IH‐induced increase in EREG expression

**Figure 5:**
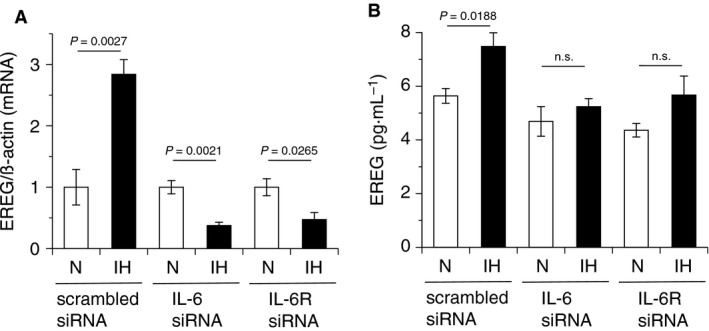
The IH‐induced increase in EREG expression was attenuated by siRNA directed against IL‐6 and the IL‐6 receptor. hCASMCs were cultured for 24 h with serum‐free medium and transfected with scrambled, IL‐6, or IL‐6 receptor siRNA. After 3‐h culture, the cells were exposed to normoxia, IH, or SH for 24 h. (A) Then, total RNA were extracted, and real‐time RT‐PCR was performed using specific primers for human EREG mRNA, as described in the Materials and methods section. Each value was normalized by arbitrarily setting the value of β–actin of the cells exposed to normoxia to 1.0. The results are representative of four independent experiments. (B) Conditioned media of normoxic‐ and IH‐treated hCASMCs were collected and used in an EREG immunoassay, as described in the Materials and methods section. IH‐induced increase in EREG was attenuated by IL‐6 and IL‐6R siRNA as well as mRNA level. The results are representative of three to four independent experiments. Each point represents the mean ± SEM. ‘n.s.’, not significant.

Taking into account the IH‐induced increase in IL‐6 (Fig. 3) and IL‐6‐induced upregulation of EREG mRNA (Fig. 4) in hCASMCs, we hypothesized that elevated IL‐6 production due to IH was responsible for the IH‐induced increase in EREG mRNA (Fig. 1). To determine the direct role of IL‐6 in IH‐induced increases in EREG mRNA expression, we applied the RNAi method using siRNA for IL‐6 and the IL‐6 receptor. As shown in Fig. 5A, both siRNA for IL‐6 and the IL‐6 receptor significantly suppressed the IH‐induced increase in EREG mRNA expression, whereas IH increased EREG mRNA in scrambled RNA had been introduced cells. We additionally investigated the direct involvement of the IL‐6/IL‐6 receptor system in IH‐induced increase in EREG production using ELISA assay. As shown in Fig. 5B, IH induced an increase in EREG in the conditioned medium of scrambled siRNA‐treated cell, whereas the IH‐induced increase in EREG in the conditioned medium of IL‐6 or IL‐6 receptor siRNA(s)‐treated cell was attenuated. These results indicated that the IL‐6/IL‐6 receptor system was important in the IH‐induced increase in EREG production in VSMCs.

## Discussion

Patients with OSA experience repetitive episodes of IH during sleep (i.e., transient desaturation and resaturation of blood oxygen) 10. IH is associated with high morbidity and mortality due to cardiovascular events 10. Atherosclerosis, which is a major cause of cardiovascular events, progresses via several steps, including the disruption of the endothelial barrier, foam‐cell formation, and excessive proliferation of VSMCs 7. In the previous study using RASMCs, we reported that IH induced the proliferation of RASMCs through upregulation of members of the EGF family and erbB2 receptor 1, suggesting that IH plays a direct role in the progression of atherosclerosis. However, the mechanisms underlying how IH increases the expression of the EGF family remained unclear. In this study, we used hCASMCs instead of RASMCs to investigate the mechanisms underlying IH‐induced upregulation of EREG mRNA. We aimed to elucidate the general mechanisms of atherosclerosis progression facilitated or induced by OSA, as well as species differences in cellular responses that are frequently observed in various tissues and cells 27, 28, 29. In our previous study, among members of the EGF gene family, EREG mRNA expression showed the most increase in response to IH, and an increase in EREG in the IH‐exposed RASMC conditioned medium was also observed 1. Thus, to determine whether the cellular responses of RASMCs to IH were similar to those observed in hCASMCs, we evaluated EREG expression in hCASMCs. We found that IH but not SH induced upregulation of EREG mRNA, pro‐EREG, and EREG in hCASMCs (Fig. 1). In addition, AREG mRNA, a significant increase of which was observed in RASMCs in response to IH, also increased in hCASMCs exposed to IH (data not shown). These results were similar to the findings of our previous study using RASMCs 1. They suggested that IH‐induced increases in EREG and AREG mRNA expression are a general feature of VSMCs. Thus, increases in EREG and AREG productions could be an important response of VSMCs to IH. On the other hand, the EREG promoter was not activated by IH stimulation (Fig. 2), suggesting that IH‐induced gene expression of EREG was not directly regulated by transcription.

Recent studies revealed that serum IL‐6 levels were elevated in patients with moderate and severe OSA after sleep 16, 17, 18. IL‐6 is a well‐known inflammatory cytokine, which has been linked to atherosclerosis 30, 31, 32, 33. Significantly higher levels of IL‐6 were detected in human arterial atherosclerotic wall plaques and carotid artery plaques 34, 35. In the present study, mRNA levels of IL‐6 in hCASMCs significantly increased in response to IH. As previous reports suggested that the IL‐6 promoter was activated by nuclear factor‐kappa B (NFκB) 36, 37 and that NFκB activation was induced by IH 15, 38, 39, it is reasonable to speculate that IH elicited IL‐6 mRNA. As shown in Fig. 3A,B, IL‐6 mRNA was significantly increased by IH. In addition, IL‐6 mRNA was elevated in RASMCs (data not shown). Furthermore, mature IL‐6, as well as IL‐6 mRNA, increased in a time‐dependent manner in IH‐exposed hCASMC conditioned media (Fig. 3C). These results suggest that IH‐induced increases in IL‐6 production are a general phenomenon of VSMCs in response to IH, regardless of species. IH‐induced increases in IL‐6 levels point to a potential role for IL‐6 in IH‐induced progression of atherosclerosis. IL‐6 makes a complex with soluble IL‐6 receptor α and recruits monocytes to areas of inflammation via the production of chemokine (C–C motif) ligand 2 in endothelial cells, which is histologically observed in chronic inflammation 40. In addition, IL‐6 elicits two major scavenger receptors, scavenger receptor‐A and CD36, in mouse macrophages, which mediate the uptake of cholesterol and make foam cells 41. In a meta‐analysis of 29 population‐based prospective studies, IL‐6 was associated with an increase in the adjusted relative risk for nonfatal myocardial infarction or coronary heart disease death 42. The present study is the first to report that IH enhanced IL‐6 production in an *in vitro* IH model, which is consistent with clinical reports 16, 17, 18. In this study, both siRNA for IL‐6 and IL‐6 receptor suppressed EREG mRNA expression much lower than that in normoxia, as shown in Fig. 5A. However, IL‐6 and IL‐6 receptor siRNA‐derived excessive suppression of IH‐induced EREG production was not observed (Fig. 5B). We do not know why IL‐6 or IL‐6 receptor siRNA‐induced excessive suppression of EREG mRNA occurred, but thought that IH may influence directly on IL‐6‐related transcriptional and/or translational activities for EREG. This matter needs further investigation.

Epiregulin is well known to possess a range of functions in normal physiological states, as well as in pathological conditions 43. It contributes to various processes, such as inflammation, tissue repair, and wound healing, by regulating angiogenesis and stimulating cell proliferation 43. A number of studies reported that the IL‐6 amplifier, which acts as a chemokine inducer in nonimmune cells, simultaneously activated NFκB and signal transducer and activator of transcription 3 to induce cytokines, such as IL‐6, and locally attracted various immune cells 44, 45, 46. Recently, the involvement of EREG in the potentiation of the IL‐6 amplifier was reported 19. These reports suggest that IL‐6 and EREG cooperatively induce dysregulation of local homeostasis via inflammatory progression. As shown in Figs 4 and 5, our results suggested that IL‐6 mediated the IH‐induced increase in EREG mRNA expression and EREG production in hCASMCs. Taken together, our results and those of previous studies suggest that dysregulation of local homeostasis via an inflammatory reaction, which is induced by IL‐6 and EREG, might also occur in patients with OSA and cause consequent OSA‐related diseases.

## Author contributions

This work was carried out in collaboration between all authors. YK and ST designed the study; acquired, analyzed, and interpreted the data; and drafted most of the manuscript. AI, AY, SS, and MM developed research questions and revised the manuscript. MY revised the manuscript.
